# From microfluidics to nanodelivery: artificial intelligence reshapes neuropharmacology research strategies

**DOI:** 10.3389/fphar.2026.1796070

**Published:** 2026-03-25

**Authors:** Chunbao Chen, Xue Du, Jing Yang, Yong Zeng, Hongwei Xie, Daihua Wang, Pingqiang Qi, Yijun Zeng

**Affiliations:** 1 Department of Neurosurgery, The 3RD Affiliated Hospital of Chengdu Medical College, Chengdu Pidu District People’s Hospital, Chengdu, China; 2 Department of Oncology, The 3RD Affiliated Hospital of Chengdu Medical College, Chengdu Pidu District People’s Hospital, Chengdu, China; 3 Department of Surgery, Nanchong Maternity and Child Health Hospital, Nanchong Paediatric Hospital, Nanchong, China

**Keywords:** artificial intelligence, drug screening, micro/nanotechnology, microfluidics, neuropharmacology, personalized therapy

## Abstract

Neurological diseases are characterized by complex etiology, heterogeneity, and significant differences in therapeutic responses, which severely constrain the efficiency of new drug development and the success rate of clinical translation. In recent years, micro- and nanotechnology (micro/nanotechnology) technologies have made breakthroughs in *vitro* disease modeling, drug delivery, and high-throughput screening, while artificial intelligence (AI) has shown strong advantages in big data analysis, pattern recognition, and predictive modeling, and the deep integration of the two has provided a new technological paradigm for neuropharmacology research. In this review, we systematically review the key applications of micro- and nanotechnology in neuropharmacology, including microfluidic brain chips, nanodelivery systems, and multiscale biosensing platforms, and focus on the central role of AI in drug screening, efficacy assessment, and personalized therapeutic decision-making.

## 1 Introduction

Neurological disease burden analysis shows that 3.4 billion people will suffer from neurological disorders globally in 2021, affecting 43% of the global population. Different types of neurological disorders ranging from migraine to stroke, and parkinsonism and dementia are now the number one cause of the global burden of disease (GBD, 2021 Nervous System Disorders Collaborators, 2024). However, due to the highly complex structural and functional properties of the nervous system, the success rate of traditional drug development models in the neurological field is significantly lower than in other disease areas (Hay et al., 2014). The blood-brain barrier (BBB) strictly restricts drugs from entering the brain, and more than 98% of small molecules and the majority of macromolecular therapies cannot be effectively delivered to the target (Ballabh et al., 2004), animal models are difficult to simulate the complex pathology and advanced cognitive functions of human neurological disorders, which leads to a disconnect between preclinical data and the results of human trials (Seyhan, 2019), and the huge differences in the patient’s response to drugs due to genetic, environmental, and disease heterogeneity make clinical trial design difficult and efficacy difficult to generalize (Selby et al., 2025). These bottlenecks not only result in a higher failure rate of clinical trials, but also prevent many promising therapeutic strategies from reaching the laboratory stage. Confronted with this dilemma, the revolution of research paradigm is imminent. *In vitro* biomimetic models represented by microfluidic organ chips (Augustine et al., 2021) and precision therapeutic tools centered on smart nano-delivery are reshaping the research pathway from both disease simulation and treatment implementation. Microfluidics is revolutionizing the drug discovery and development process with its advantages of miniaturization, high throughput, and precise fluid manipulation, providing the pharmaceutical industry with an efficient platform from target screening to formulation development (Krishna Kis et al., 2025).

In the technological evolution from microfluidics to nano-delivery, the role of AI has been upgraded from an auxiliary tool to a core decision engine. At the model construction side, AI drives the intelligent microfluidic system to transcend static culture (Fergola et al., 2025), and through analyzing high content imaging (HCI) and multi-omics data (Moen et al., 2019), autonomously optimize cell combinations (Chen et al., 2025), fluid shear and biochemical gradients (Yu et al., 2024), dynamically simulate neuroinflammation (Lohasz et al., 2025), and the complex process of blood-brain barrier transport (Lee et al., 2022), to generate a high-fidelity digital pathology twin (Zhang et al., 2024). On the drug delivery side, AI is reshaping the rational design process of nanocarriers, with molecular dynamics simulations and deep learning predictions that can accurately plan the optimal path of nanoparticles across the BBB (Rostami et al., 2026). Using generative modeling, novel nanomaterials with ideal intracerebral distribution, controlled release properties and low immunogenicity can be designed from scratch (Khater et al., 2025). In addition, AI builds a bridge between model prediction and *in vivo* efficacy by integrating efficacy data from organ microarrays, animal experimental images, and patient genomic information to build interpretable computational pharmacology models (Mulay et al., 2024), which dramatically improves the credibility of efficacy prediction from *in vitro* to the clinic. Overall, AI is profoundly transforming pharmaceutical sciences by integrating machine learning, data-driven modeling, and algorithmic optimization, demonstrating tremendous clinical impact and application potential in areas such as drug design, delivery system development, clinical trial optimization, and personalized medicine (Halagali et al., 2025).

**FIGURE 1:**
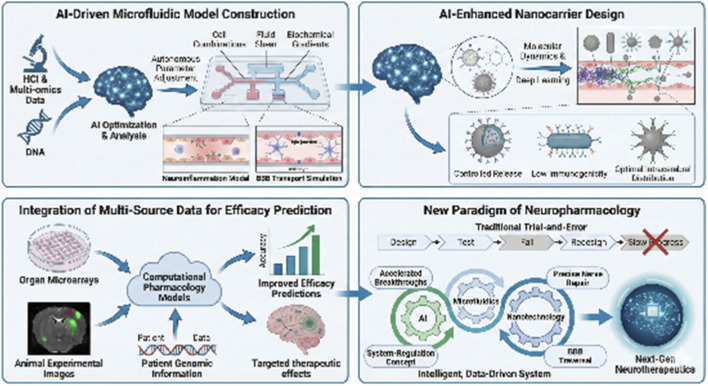
AI-Driven Microfluidic Model Construction: Show the role of AI in optimizing microfluidic systems for simulating neuroinflammation and blood-brain barrier (BBB) transport. AI-Enhanced Nanocarrier Design: Depict the use of AI in the rational design of nanocarriers, with a focus on molecular dynamics simulations and deep learning predictions to optimize nanoparticle paths across the BBB. Integration of Multi-Source Data for Efficacy Prediction: Show the integration of organ microarrays, animal experimental images, and patient genomic information to build computational pharmacology models. New Paradigm of Neuropharmacology: Illustrate the fusion of AI, microfluidics, and nanotechnology, transitioning from linear trial-and-error research to an intelligent, data-driven system. The figures in this study were made using the BioGDP online platform (https://biogdp.com/).

The triple fusion of AI, microfluidics and nanotechnology marked the birth of a new paradigm, moving away from linear research and development relying on trial and error to an intelligent, closed-loop system that is data-driven and can be iteratively optimized. It is expected to accelerate the breakthrough of the BBB and realize precise nerve repair, and more likely to promote neuropharmacology from the single target concept to the system regulation time, and to promote the paradigm shift of neuropharmacology from experience-driven to data- and model-driven. It could also push neuropharmacology from single target thinking to system regulation time, and push neuropharmacology from experience-driven to data- and model-driven paradigm shift (Figure 1).

## 2 A new paradigm for neuropathology modeling and drug screening

### 2.1 Precise construction of bionic neural microenvironments

The combination of AI and microfluidics realizes the leap from structural simulation to functional replication of neural models. The precise construction of bionic neural microenvironments is able to simulate the key processes of neuron-glia interaction, axon guidance and synaptic plasticity by integrating biomaterials, microfluidic technology and cell engineering (Yan et al., 2024; Habibey et al., 2022). This highly simulated microenvironment not only provides a more physiologically relevant model for studying the pathogenesis of neurodegenerative diseases, such as Alzheimer’s disease (AD) and Parkinson’s disease (PD) (Mofazzal Jahromi et al., 2019; Toh et al., 2023), but also reveals the effects of microenvironmental changes, such as gradients of inflammatory factors and alterations in matrix stiffness, on the survival and function of neurons in the course of the pathological process (Saliba et al., 2025; Huang et al., 2020). By modulating the mechanical properties and spatial distribution of biochemical factors of hydrogels, the microenvironment of β-amyloid deposition (Sahtoe et al., 2024) or α-synuclein aggregation (Jo et al., 2021) in brain tissues can be simulated, thus reproducing disease-specific pathological features *in vitro*.

For the simulation of dynamic pathological processes, a recently developed machine learning-driven microfluidic electrophysiology platform provides a breakthrough tool for dynamic simulation of neuropathological processes (Xu et al., 2026). The platform deeply combines a high-throughput microfluidic chip with a multi-electrode array (MEA), and introduces a dual-channel long short-term memory (LSTM) deep learning network, which realizes dynamic, real-time decoding of electrical signal interactions between glioma cells and neurons. This study also successfully captured the key electrophysiological features of nerve-tumor crosstalk in the tumor microenvironment, and the system was able to automatically identify the “hijacking” patterns of glioma cells on nerve signals. For the first time, the system has revealed the specific electrical activity patterns of tumor-affected neural circuits *in vitro*, which establishes a high-precision and intelligent research methodology platform for the in-depth analysis of the dynamic mechanisms of neural-related diseases. In addition, by constructing disease-specific neural microenvironments, researchers were able to assess the regulatory effects of drugs on neuronal protection, synaptic function repair, and glial cell activation *in vitro* (Xu et al., 2021; Habanjar et al., 2021), as well as to examine the permeability and distribution patterns of drugs in complex three-dimensional environments (Vescio et al., 1987).

### 2.2 Microfluidics and brain-like models in drug screening

Microfluidic chips can precisely regulate cell growth conditions and chemical gradients in a micro-scale environment, providing a highly controllable platform for modeling neurological diseases (Kilic et al., 2016). Microfluidic-based brain-on-a-chip can reconstruct neuron-glia interactions, synaptic connections, and blood-brain barrier structure, which significantly improves the prediction of clinical efficacy of *in vitro* models (Park et al., 2015). Combined with patient-derived induced pluripotent stem cells (iPSCs), the microfluidic platform can be used to construct individualized neurological disease models, laying the foundation for precise drug administration and efficacy assessment (Vatine et al., 2019).

The neurovascular-unit-on-a-chip, which integrates the BBB function, allows real-time assessment of the efficiency of drug candidates in penetrating the BBB and the impact on the barrier integrity, and effectively screens out compounds that are effective *in vitro* but do not enter the brain (Augustine et al., 2021; Wevers et al., 2018). With the help of HCI, microelectrode arrays and multi-omics analysis of supernatants, researchers can assess the effects of drugs on multidimensional functional indicators of neuronal survival, axon guidance, synaptic transmission, glial inflammatory response, and network oscillatory activity in parallel on the same platform (Smirnova and Hartung, 2024; Mostajo-Radji et al., 2020; Trujillo et al., 2019). For example, in a brain chip model of amyotrophic lateral sclerosis (ALS), multiple modulatory effects of drugs on motor neuron survival, astrocyte toxicity, and neuromuscular junction function can be observed simultaneously (Osaki et al., 2018; de Jongh et al., 2021). This multi-parameter, functional screening in an approximate *in vivo* physiological environment generates richer and more predictive datasets (Yamazaki and Ishida, 2025), which can more reliably identify promising lead compounds at the preclinical stage, optimize dosing strategies, and dramatically reduce costly late-stage drug depletion due to preclinical model failures.

### 2.3 Multi-scale biosensing and neural signal monitoring

By integrating advanced sensing technologies such as high-density microelectrode arrays, fiber-optic calcium imaging, and wearable neural probes (Steinmetz et al., 2019; Weisenburger and Vaziri, 2018; Jun et al., 2017), AI is able to collect massive spatiotemporal dynamic data in real time, ranging from single neuron discharges to large-scale network oscillations. Deep learning models, in particular convolutional neural networks and recurrent neural networks, are able to automatically identify specific neural coding patterns in response to pharmacological interventions (Mathis et al., 2018), such as temporal features of dopaminergic signals (Dabney et al., 2020), power changes of gamma oscillations (Abbaspoor et al., 2023) or reconstruction of synchronization across brain regions (Zhou et al., 2025). Unsupervised learning allows for the discovery of changes in neural representations outside the predefined framework of human researchers, revealing unknown mechanistic pathways of drug action (Pandarinat et al., 2018). This data-driven research strategy makes it possible to achieve dynamic resolution of drug effects with millisecond precision in freely behaving animals and even in patients in the future, greatly expanding the dimension and depth of neuropharmacological observations.

**FIGURE 2:**
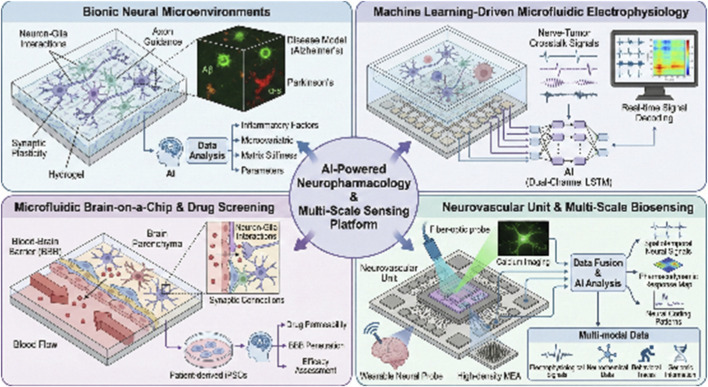
Precise Construction of Bionic Neural Microenvironments: Visualize the integration of AI, microfluidics, and cell engineering to create bionic neural microenvironments that simulate neuron-glia interactions, axon guidance, and synaptic plasticity. Machine Learning-Driven Microfluidic Electrophysiology Platform: Illustrate a microfluidic electrophysiology platform integrated with a multi-electrode array (MEA) and a dual-channel LSTM deep learning network. Microfluidic Brain-on-a-Chip for Drug Screening: Visualize a microfluidic brain-on-a-chip that mimics neuron-glia interactions, synaptic connections, and blood-brain barrier (BBB) structure. Neurovascular-Unit-on-a-Chip for BBB Functionality and Drug Penetration: Create a schematic showing a neurovascular-unit-on-a-chip model for assessing drug candidates’ ability to cross the BBB and impact barrier integrity.

AI-driven multimodal data fusion is building a complete chain of pharmacodynamic assessment from molecular to behavioral. While there are inherent limitations in the data generated by a single technology platform, AI is able to integrate electrophysiological signals, neurochemical sensor data, behavioral video traces, and genomic information to build a unified pharmacodynamic response map (Arkhipov et al., 2025; Zhao et al., 2021; Ma et al., 2025). The dynamic coupling between neuronal activity, local field potentials and the concentration of specific neurotransmitter release can be established by graph neural network (GNN), which can accurately quantify the intensity of drug modulation of neural circuit function (Bessadok et al., 2023; Betzel and Bassett, 2017). Migration learning can enhance the predictive value of preclinical models by migrating effective features obtained in animal models to the MEA data parsing of human-derived organoids or brain microarrays (Brynildsen et al., 2023; Buccino et al., 2018). Ultimately, these multi-scale biomarkers mined and correlated by AI can not only distinguish the therapeutic effects and side effects of drugs more accurately, but also provide an objective typing basis based on neurophysiological characteristics for individualized medication, and promote a paradigm shift in the treatment of neuropsychiatric disorders from symptomatic relief to loop repair (Figure 2).

## 3 Breaking the blood-brain barrier and targeted regulation

### 3.1 Design and optimization of nanocarriers

AI is enabling rational design and full chain optimization of nanocarriers by integrating material physicochemical properties, physiological barrier characteristics and molecular interactions data. Based on deep generative models such as variational self-encoders and generative adversarial networks, researchers can efficiently explore the synthetic sequences of massive polymers, lipids and inorganic materials in virtual chemical space and automatically predict key parameters such as their self-assembly behaviors, drug-carrying capacity and stability (Wu et al., 2017; Joshi and Kumar, 2021; Atas Guvenilir and Doğan, 2023). Meanwhile, GNN are able to model the multi-scale interactions between chemical groups on the surface of nanoparticles and receptors on the surface of endothelial cells of the blood-brain barrier, and accurately predict their transcellular transport efficiency and intracellular release kinetics (Wu et al., 2023; Wang DD. et al., 2024). This data-driven design paradigm enables the development of smart nanocarriers with spatiotemporally controllable drug release and lesion microenvironment response properties (Sanchez-Lengeling and Aspuru-Guzik, 2018), providing a novel engineering solution for breaking through the BBB.

AI-enabled nanocarrier optimization further focuses on individualized adaptation and precise regulation of *in vivo* fate. By integrating clinical images (e.g., BBB permeability region mapping), patient multi-omics data (e.g., BBB-associated protein expression profiles), and animal experimental validation data through migration learning, AI can construct a patient-carrier matching model to recommend optimal carrier materials, particle sizes, and surface modification strategies for different types of diseases or individual differences (Girigoswami and Girigoswami, 2025; Mazumdar et al., 2025). At the kinetic level, reinforcement learning algorithms are able to simulate the trajectory of nanoparticles in complex vascular networks and their interactions with biological barriers, and reverse-optimize their morphology, stiffness, and surface charge to maximize their enrichment at the lesion site (Zhu et al., 2025; Shan et al., 2024). In addition, by integrating real-time biosensing data (e.g., intracranial microdialysis and near-infrared imaging), AI is able to construct closed-loop feedback systems (Scangos et al., 2021) and dynamically regulate the release rate and targeting of carriers to achieve on-demand precision drug delivery to neural circuits or specific cell populations (Mitchell et al., 2021), elevating the precision of neuropharmacological interventions to an unprecedented level.

### 3.2 Real-time tracking and feedback regulation of the delivery process

Based on the fusion of multimodal *in vivo* imaging technology and artificial intelligence, it provides a revolutionary solution to overcome the black box state of the delivery process. By integrating advanced technologies of near-infrared two-region imaging with high spatial and temporal resolution, ultrasound localization microscopy, and magnetic particle imaging, AI is able to dynamically track nanocarriers injected *in vivo* from whole-body to single-cell scale (Miao and Pu, 2016; Liang et al., 2023; Kæstel-Hansen et al., 2025). Deep learning models, especially spatio-temporal convolutional networks, can decipher complex multidimensional image data streams in real time to accurately quantify the trajectory of carrier flow within the vasculature, the retention and intercellular processes at the blood-brain barrier, and the rate of accumulation in the lesion area (Guo B. et al., 2024; Wu et al., 2021). In addition, AI models these image features in association with synchronously recorded physiological parameters to construct a dynamic causal map of carrier fate and microenvironmental response (Ng et al., 2020), enabling global visualization and quantitative analysis of the delivery process.

**FIGURE 3:**
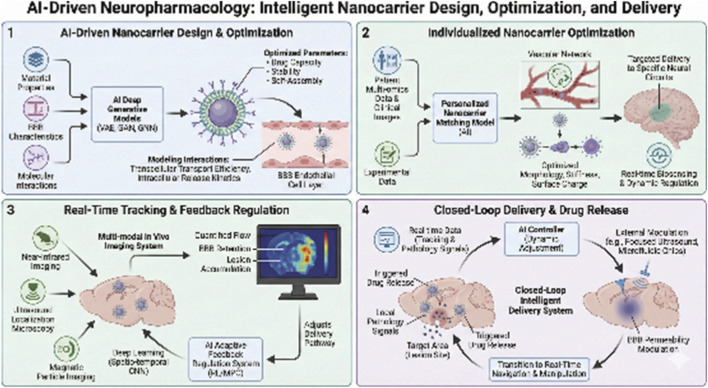
Design and Optimization of Nanocarriers: Visualize the integration of AI in the rational design and optimization of nanocarriers, focusing on material properties, physiological barrier characteristics, and molecular interactions. Individualized Nanocarrier Optimization for Drug Delivery: Show how AI uses patient multi-omics data, clinical images, and experimental data to construct a personalized nanocarrier matching model. Real-Time Tracking and Feedback Regulation of Nanocarriers: Visualize a multi-modal *in vivo* imaging system (near-infrared imaging, ultrasound localization microscopy, magnetic particle imaging) integrated with AI to track nanocarrier delivery from whole-body to single-cell scale. Closed-Loop Delivery and Drug Release: Illustrate the transition from open-loop to intelligent closed-loop delivery systems, where AI dynamically adjusts carrier behavior based on real-time data.

Based on real-time sensing, the AI-driven adaptive feedback regulation system upgrades the delivery from open-loop to intelligent closed-loop. Taking real-time imaging and biosensing data as continuous inputs, it dynamically calculates and outputs optimal regulation instructions through reinforcement learning or model predictive control algorithms (Templer, 2022; Siegel, 2014). When the system monitors that the carrier is not enriched in the target brain region as expected through imaging, it can instantly trigger an *ex vivo* focused ultrasound device to acoustically modulate specific cerebral vascular segments to transiently and reversibly increase the permeability of the blood-brain barrier and guide the carrier to efficiently pass through (Airan et al., 2017; Wang et al., 2022). Alternatively, the implantable microfluidic chip can be modulated to change the concentration of co-injected “adjuvant” molecules to adjust the surface properties and targeting of the carriers (Lee et al., 2025; Xia et al., 2024). This closed loop of monitoring-decision-regulation can complete multiple iterations in millisecond to minute time scales, ensuring that the carrier is delivered in the optimal pathway and automatically triggering drug release when local pathology signals are sensed (Gu et al., 2013; Mickle et al., 2019). This marks the transition of neuropharmacology from the traditional wait after injection model to the cruise therapy era of real-time navigation and manipulation (Figure 3).

## 4 Synergistic integration models of AI and micro/nanotechnology

### 4.1 AI-mediated linkage of microfluidics and nanodelivery

The combination of microfluidic technology and nano-delivery system provides an unprecedented controlled microenvironment for drug screening and delivery optimization, and the intervention of artificial intelligence upgrades it from static manipulation to dynamic intelligent linkage (Liu et al., 2021; Liu et al., 2024). In the traditional coupling mode, microfluidic chips are mainly used for high-throughput generation of monodisperse nanoparticles or simulation of biological barriers, and the process parameters need to be manually preset and optimized (Karnik et al., 2008). Through the introduction of AI, especially reinforcement learning and digital twin models, the system is able to achieve adaptive co-regulation, where AI analyzes the monitoring data of the nanoparticle synthesis module in the chip in real time and dynamically adjusts the micropump flow rate, mixing ratio, and reaction time in order to stably produce a carrier batch with optimal physicochemical properties (Chen and Lv, 2022; Guo K. et al., 2024). Meanwhile, in the parallel delivery simulation module, the AI optimizes the synthesis parameters based on the real-time feedback of the carrier properties and the cell/tissue barriers cultured in the chip, forming a closed-loop iterative process of preparation-test-optimization (Bhatia and Ingber, 2014). This linkage makes the development of nanocarriers no longer an isolated process, but a continuous intelligent engineering linked to the downstream biological effects in real time.

At the individualized therapeutic level, AI-mediated microfluidic-nano linkage platforms are evolving towards the patient-on-a-chip customization model. By integrating on-chip multi-omics detection units, microfluidic chips can rapidly analyze patient-derived body fluids or cellular samples to obtain key biomarker profiles (Surappa et al., 2023; Abdulla et al., 2022), such as blood-brain-barrier-associated protein expression, and tumor cell-surface receptor abundance (Ahmadi et al., 2024; Zhao et al., 2025). After parsing these data, the AI model drives the synthesis module on the chip to customize personalized nanomedicines in real time to best match the patient’s biological characteristics, including customizing target ligands, adjusting stimulus response thresholds, or optimizing drug loading ratios (Qi and Hu, 2025; Alavi et al., 2024). Further, the subsequent on-chip efficacy assessment can be seamlessly connected, where the customized nanomedicine is directly applied to patient-like organs or pathological tissue sections cultured on the same chip (Kheiri et al., 2024), and multi-dimensional efficacy data are collected in real time through high-content imaging and metabolic sensing, and efficacy prediction and dosage optimization are performed by AI (Cao et al., 2023). This integrated intelligent platform of “sample in, protocol out” deeply integrates the precise manipulation of microfluidics, the transformative power of nanotechnology and the decision-making intelligence of AI, laying a technological foundation for the realization of on-demand therapies ranging from scaled-up screening to individualized precision preparation.

### 4.2 Integration of multi-dimensional data analysis and mechanism analysis

The powerful data integration capability of AI has promoted the in-depth development of neuropharmacology mechanism research. In multi-target drug development, the AI platform integrates genomics, proteomics and microfluidic electrophysiology data, constructs a dynamic network model of neural signaling pathways, and successfully identifies a combination of key ion channel targets for glioma invasion (Geschwind and Konopka, 2009; Chen and Pesaran, 2021), which provides a mechanistic basis for multi-target nano drug design. Microfluidic chips and nanosensors are capable of synchronized acquisition of cross-scale, multimodal data streams covering ion channel currents, single-cell secreted protein profiles, neurotransmitter dynamic concentrations, cytoskeletal mechanical changes, and real-time trajectories of nanocarriers with unprecedented spatial and temporal resolution (Wang M. et al., 2024; Choi et al., 2021; Kutluk et al., 2024). Through GNN, AI is able to map data of different dimensions into a unified biological network model, automatically identify complex causal chains, and reveal time-lagged influences and feedback loops between signals of different dimensions (Zhang et al., 2022; Sanchez-Romero and Cole, 2021).

**FIGURE 4:**
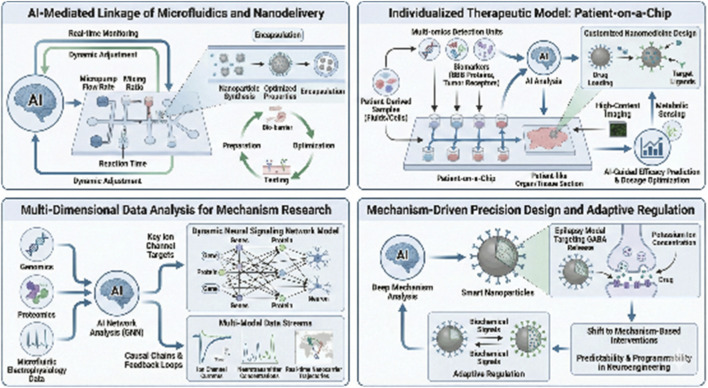
AI-Mediated Linkage of Microfluidics and Nanodelivery: Visualize the integration of microfluidic technology and nanocarrier delivery systems, showing how AI drives adaptive co-regulation. Individualized Therapeutic Model: Patient-on-a-Chip, depict the AI-driven, microfluidic-nano linkage platform used for personalized nanomedicine design. Multi-Dimensional Data Analysis for Mechanism Research: Visualize the integration of genomics, proteomics, and microfluidic electrophysiology data to construct a dynamic neural signaling network model. Mechanism-Driven Precision Design and Adaptive Regulation: Show how AI empowers the design of smart nanoparticles based on deep mechanism analysis, such as targeting GABA release in epilepsy models.

Based on deep mechanism analysis, AI further empowers micro/nano-systems to realize mechanism-led precision design and adaptive regulation. AI-driven mechanism analysis enables the design process to be anchored to specific biophysical or biochemical pathway nodes, and through analysis, it is found that the reduced release probability of synaptic vesicles of the epileptic model Gama-aminobutyric acid (GABA) is a major defect. GABA synaptic vesicle release probability was found to be a major defect in the epilepsy model, AI can guide the design of smart nanoparticles modified with a specific presynaptic membrane targeting peptide and capable of releasing GABA reuptake inhibitors in response to changes in the local potassium ion concentration (Rhys et al., 2022; Naskar et al., 2021). At the regulatory level, the system no longer only responds to phenotypic signals, but can carry out combinatorial and hierarchical precise interventions based on the mechanism status analyzed by AI in real time (Paz et al., 2013). This paradigm upgrade from symptom response to mechanism repair signifies that neuropharmacology and neuroengineering are entering a new era of predictability, interpretability, and programmability, and lays the methodological foundation for the realization of curative interventions for neurological diseases (Figure 4).

## 5 Challenges and future prospects

The integration of artificial intelligence and micro/nanotechnology is promising, but its development still faces multiple serious challenges. First, the data barrier is the core bottleneck, as the high-dimensional multimodal data generated by microfluidic and nanosensors are in different formats, lack of unified standards, and involve a large amount of undisclosed “dark data”. Secondly, the issue of model interpretability is particularly critical in the life science field. Complex deep learning models are often regarded as “black boxes”, and the mechanisms or design rules derived from the data are not supported by clear biophysical or biochemical principles, making it difficult to trust the biological community and guide reliable research. The mechanisms or design rules deduced from the data are not supported by clear biophysical or biochemical principles, which makes it difficult to convince the biological community and guide reliable experimental verification. In addition, the huge gap from *in vitro* microarrays to *in vivo* applications poses another challenge.

In the future, breakthroughs in this field will depend on the synergistic evolution of three major directions. First, the establishment of a standardized intelligence experiment platform, through the development of data and interface standards, the development of open-source, modular “brain-chip” and intelligent nano-synthesis platforms, so that AI algorithms can continue to learn and optimize on the comparable data generated by different laboratories. The first is “causal AI”, which is a community-driven iterative innovation. Second, the deep integration of “causal AI”, the next-generation of AI tools will go beyond pattern recognition and focus on discovering the causal structure between variables. Ultimately, this will all converge in an end-to-end personalized medicine closed loop where patient imaging and molecular diagnostic data can be fed into an AI platform in real-time, driving a microfluidic system at the bedside or in the clinic to rapidly synthesize and test personalized nanomedicines. Once treatment is initiated, implantable nanosensors and external imaging will continuously monitor efficacy and microenvironmental changes to form a real-time treatment optimization loop. This will completely transform the treatment from a “one-size-fits-all” reactive model to a new paradigm of data-driven, dynamically adjusted precision medicine that aims at eradicating diseases, and ultimately realizing precise repair and functional reconstruction of diseases from the molecular to the behavioral level.

## 6 Conclusion

Through empowering microfluidics for bionic modeling and high-throughput screening, and optimizing the targeting and controllability of nano-delivery systems, AI is reshaping the research paradigm of neuropharmacology. The depth of the three breaks through the technical bottleneck of traditional research, realizing the whole chain of innovation from pathology simulation to drug development. Despite the challenges of model generalization, data standardization and interpretability, with the continuous iteration of technology, AI will become the core driving force for the development of precision and personalization in neuropharmacology, bringing revolutionary breakthroughs in the treatment of central nervous system diseases.
